# Bridging the Gap: Identifying and Overcoming Barriers to CAR-T Access Through Streamlined and Standardized Pathways

**DOI:** 10.1016/j.mayocpiqo.2025.100693

**Published:** 2026-01-05

**Authors:** Kanishka Uttam Chandani, Nandita Khera

**Affiliations:** From the Division of Hematology Oncology, Mayo Clinic, Phoenix, AZ

Cell and gene therapies (CGTs), including chimeric antigen receptor (CAR) T-cell therapy, represent a transformative advancement in the treatment landscape for not only hematologic malignancies but also various solid tumors and rheumatological, and neurological conditions.^1^ However, despite the Food and Drug Administration approval of 46 CGTs,^2^ equitable access remains limited by a range of systemic and institutional barriers.^3^ These include capacity constraints, clinical and operational complexity, and complex reimbursement mechanisms, which unveil the disparities between well-resourced centers of excellence, which often operate with prenegotiated CAR-T contracts, and other institutions that must navigate by case-by-case financial agreements. Additionally, the geographical distribution of these centers of excellence offering life-saving CAR-T treatment is skewed, which leads to certain states being classified as poor access states with an average distance to a treatment center offering CAR-T therapy being over 100 miles.^4^ The compound effect of these socioeconomic and institutional biases not only restricts this potentially life-saving treatment to a much smaller population but also causes tangible delays in delivering disease-modifying therapies, thus leading to poorer patient outcomes.^5^ As we advance the science of medicine, we must focus on addressing the parallel challenge of building a health care ecosystem that delivers innovation sustainably and equitably. In this issue of *Mayo Clinic Proceedings*, Singh et al^6^ provide a timely and multidisciplinary perspective from a panel of diverse stakeholders on identifying and potentially improving the barriers to CAR-T therapy. Their discussion draws upon a range of clinical, operational, and real-world payer experiences to highlight the multifaceted barriers that continue to restrict equitable access to CAR-T and other CGTs and potential ways to address them. A notable concern raised during the panel discussion was the surprising lack of standardization across treatment centers, despite the remarkable pace at which the Food and Drug Administration is approving CGTs.

The process of receiving cellular therapy can be broadly divided into 2 phases: the variable early phase—which includes patient identification/referral, informed consent, benefits verification, and financial approvals—and a more standardized phase consisting of apheresis, lymphodepletion, cell infusion, and subsequent close follow-up for toxicity monitoring and disease progression. While the latter phase has become more streamlined over the years, the former remains a bottleneck characterized by inconsistent practices and avoidable delays. This lack of harmonization represents an integral opportunity to improve system efficacy and, more importantly, to break down barriers that limit access based on institutional capacity or payer dynamics. There is also the need for raising awareness about CAR-T and its evolving indications among the referring providers, standardizing criteria, and leveraging artificial intelligence/machine learning models to identify patients who would potentially benefit from these treatments to get the process started early.

A vision emphasized by the authors is expanding the CGT delivery capacity beyond the boundaries of academic centers by streamlining the process of accreditation to enable more institutions, including community-based and regional hospitals, to administer these therapies through collaboration between newer and established centers. However, the rigorous and resource-intensive accreditation process demands a significant array of resources, including investments in time, multidisciplinary staffing, and infrastructure. These barriers are especially difficult for smaller or low-resource centers to overcome as a result of which only 159 hospitals in the United States are accredited by Foundation for the Accreditation of Cellular Therapy.^7^ The authors argue, rightly, that eliminating the redundancy in accreditation for each new CGT could significantly accelerate access. This is in concordance with the American Society for Transplantation and Cellular Therapy (ASTCT) 80/20 Taskforce that put forward potential strategies to eliminate duplicative onboarding, auditing, and logistical requirements, to aid with increasing access and scalability. The ASTCT proposal was to standardize and streamline 80% of the onboarding process for all CGTs, define expectations for toxicity monitoring to potentially replace product-specific risk evaluation and mitigation strategy programs, and thus channel the current risk evaluation and mitigation strategy education, testing, and data reporting. It also proposed unifying information technology platforms and encouraged CGT manufacturers to adopt a more consistent nomenclature.^8^

Singh et al^6^ build on this work by highlighting and offering recommendations to address critical yet often underappreciated obstacles to timely access to CGTs such as the prior authorization process. Prior authorization remains necessary for verifying coverage and coordinating care, but the lack of uniformity across centers and payers renders it highly variable and inefficient. Furthermore, the need for irrelevant testing by payers leads to significant delays in receiving treatment. These time lapses are not merely administrative inconveniences; they represent critically missed opportunities for patients with aggressive disease biology. In a survey study, 65% community oncologists noted patient deterioration before CAR-T administration owing to the rate-limiting step of payer approval processes.^9^ Singh et al^6^ advocate for improved alignment between payers and providers through standardized clinical criteria, dedicated case management teams, and the possibility of evaluation-based multiple preliminary approvals for all treatment options (hematopoietic cell transplantation vs CAR-T) being considered in the early stage. The ASTCT Committee on Practice Guidelines is working on an initiative to develop consensus-based recommendations for pre-CART evaluation to help reduce non–value-added testing, minimize delays in therapy, and improve equitable access to these potentially curative treatments (P. Carpenter and M. Hamadani, personal communication).

The authors also report on challenges and wide variance in time for financial clearance and contracting, depending on established vs newer centers and centers that treat out-of-state patients with different Medicaid agencies, for example, with some states covering CAR-T only in inpatient or outpatient settings but not both.^10^ The type of insurance and whether the hospital is in-network further complicates the speed and feasibility of access. In time-sensitive scenarios such as those involving relapsed or refractory disease, the delay caused by individualized financial negotiations can directly impact survival outcomes. The authors’ proposal for standardized and preset financial agreements is compelling, especially when lives are quite literally on the line.

An integral but often underappreciated aspect of CAR-T therapy is the associated financial toxicity associated with it, which also affects access adversely. A recent study reported that mere 7.3% of CAR-T cell therapy–related admissions were of patients from neighborhoods with a mean income of less than $40,000.^11^ Novartis, Kite/Gilead, and Bristol Myers Squibb have launched assistance programs to support eligible low-income patients, but there is lack of widespread awareness about them among all providers who come in contact with these patients.^12^

Although the article by Singh et al^6^ offers a critical contribution, it is not without limitations. The small panel size (6 participants) decreases generalizability, and perspectives from community oncologists, patient advocates, and those directly impacted by access disparities were notably absent. These voices must inform future policymaking to lead to a more solution-driven discussion.

In conclusion, expanding the clinical promise of CAR-T and other CGTs must be matched by a commitment to equitable access, unhindered by geographical boundaries, institutional biases, or income barriers. The therapeutic promise of CAR-T and other CGTs is unquestionable but fulfilling that promise equitably demands coordinated action by various stakeholders. Addressing these challenges through a multipronged coordinated strategy across clinical, regulatory, and financial domains is not optional; it is a moral and clinical imperative.

## Potential Competing Interests

Given her role as Editorial Board Member, Dr Khera was not involved in the peer-review of this article and has no access to information regarding its peer-review. The other authors report no competing interests.
